# Indonesian presidential election sentiment: Dataset of response public before 2024

**DOI:** 10.1016/j.dib.2023.109993

**Published:** 2023-12-19

**Authors:** Asno Azzawagama Firdaus, Anton Yudhana, Imam Riadi, Mahsun

**Affiliations:** a Master Program of Informatics, Universitas Ahmad Dahlan, Yogyakarta 55166, Indonesia; b Department of Electrical Engineering, Universitas Ahmad Dahlan, Yogyakarta 55166, Indonesia; c Department of Information System, Universitas Ahmad Dahlan, Yogyakarta 55166, Indonesia; d Department of Indonesian Language and Literature Education, Universitas Mataram, Mataram 83125, Indonesia

**Keywords:** Indonesia presidential candidates, Political parties, Python, Sentiment analysis, Twitter

## Abstract

Indonesia is one of the countries that is currently entering the political year for the election of President, Regional Heads, and Members of the Legislative in 2024. This has become a hot topic on social media, especially about the Presidential Election. Twitter is one of the platforms with the largest users in Indonesia. It is interesting to see the alignment of Twitter users towards presidential candidates who already have a carrying party, namely Ganjar Pranowo, Prabowo Subianto, and Anies Baswedan based on a sentiment analysis approach. User feedback data about Indonesian Presidential candidates are obtained from the Twitter platform using Twitter API with Python programming language. The data obtained was 30,000 data with each candidate as many as 10,000 data. Data is pulled in April 2023 with specific keywords. The time for data withdrawal is chosen based on the announcement of Presidential Candidates carried by political parties before the schedule for determining or campaigning for Presidential candidates. Current data can potentially be used again as a comparison of analysis of presidential candidates on campaign time spans and after campaigns or actual calculation results. The data that can be accessed is in CSV format and has gone through several stages such as labelling using Language experts, removing spam Tweets & empty cells and preprocessing.

Specifications Table

**Table:** 

Subject	Social Sciences.
Specific subject area	Data mining and text analytics on Twitter posts for collecting public opinions and social networks related to the Indonesian Presidential 2024.
Data format	Mixed Data (Raw, Analyzed, Filtered).
Type of data	Table (CSV).
Data collection	Total data: 30,000 data tweets discussing the 2024 President of Indonesia. Three potential candidates by research [1], namely Ganjar Pranowo (Governor of Central Java), Prabowo Subianto (Chairman of the Gerindra Party), and Anies Baswedan (Governor of DKI Jakarta 2017-2022). Each candidate took data from as many as 10,000 tweets with keywords: “Ganjar President Indonesia 2024”, “Prabowo President Indonesia 2024”, and “Anies President Indonesia 2024”. Data is obtained using Twitter API of Visual Studio Code tools. The data is then preprocessing using case folding, tokenizing, stop word removal, normalization, and stemming techniques.
Data source location	Country: Indonesia.
Data accessibility	Repository name: Mendeley Data Data identification number: 10.17632/7w5zvr8jgp.5 Direct URL to data: https://data.mendeley.com/datasets/7w5zvr8jgp/5
Related research article	-

## 1 Value of the Data


 

• This dataset is useful for policymakers, pollsters, political and government academics, candidates and winning teams, and political parties.
• This dataset provides insight into the fact that the Indonesian's involvement in freedom of speech on social media platforms over political issues, such as Indonesian presidential candidates will be able to validate the predictions of presidential election results through academic studies.
• This dataset can be served as a reference for future research when a candidate's campaign time starts and after the actual results of the 2024 Indonesian Presidential Election (Example: [2]). So this approach model can be proposed as another option for predicting presidential election results because it is considered better than traditional polling [3].

## 2 Background

The obtained data serves as a comparison material with other case data for the projection of the Indonesian Presidential election in 2024. For example, The obtained data could be compared with the results of obtained data during the determination of presidential candidates and throughout the campaign period, as well as the actual election results for the Indonesian Presidential election in 2024. Therefore, through a sentiment analysis approach, this research can provide reliable recommendations for projecting similar Presidential election outcomes in Indonesia or other countries.

## 3 Data Description

This dataset contains Tweet information about the discussion of the 2024 Indonesian Presidential candidate. The candidate dataset used was Ganjar Pranowo, Prabowo Subianto, and Anies Baswedan from October 2022 to April 2023. The withdrawal of data this time was taken because the three candidates were already carried by one party, such as Ganjar Pranowo is carried by the PDI-P party (Indonesian Democratic Party of Struggle), Prabowo Subianto is carried by the Gerindra Party (Great Indonesia Movement), and Anies Baswedan is carried by the Nasdem Party (National Democratic). However, only the PDI-P party is qualified to carry a presidential candidate because it is enough to win seats in the national parliament. However, this Indonesian presidential election is important because President Jokowi is going to end his two terms. PDI-P as Jokowi's party again carries a new candidate in the 2024 election, namely Ganjar Pranowo who is currently the Governor of Central Java. In addition, Prabowo Subianto, who was also Jokowi's minister until now, is running as a presidential candidate in 2024. This selection is his fourth candidacy in the Indonesian Presidential Election contestation, namely in 2009 as a Vice Presidential candidate, 2014 as a Presidential candidate, and 2019 as a Presidential candidate. Another candidate, Anies Baswedan had also been a minister in 2014-2016 during Jokowi's era as President. The data for each candidate is in a folder such as original data, labeled data, and cleaned data with each folder containing files specific to the candidate's name, such as “Anies Baswedan,” “Ganjar Pranowo,” and “Prabowo Subianto,”.

a. original data (Data Folder: original data)

**Fig. 1:**
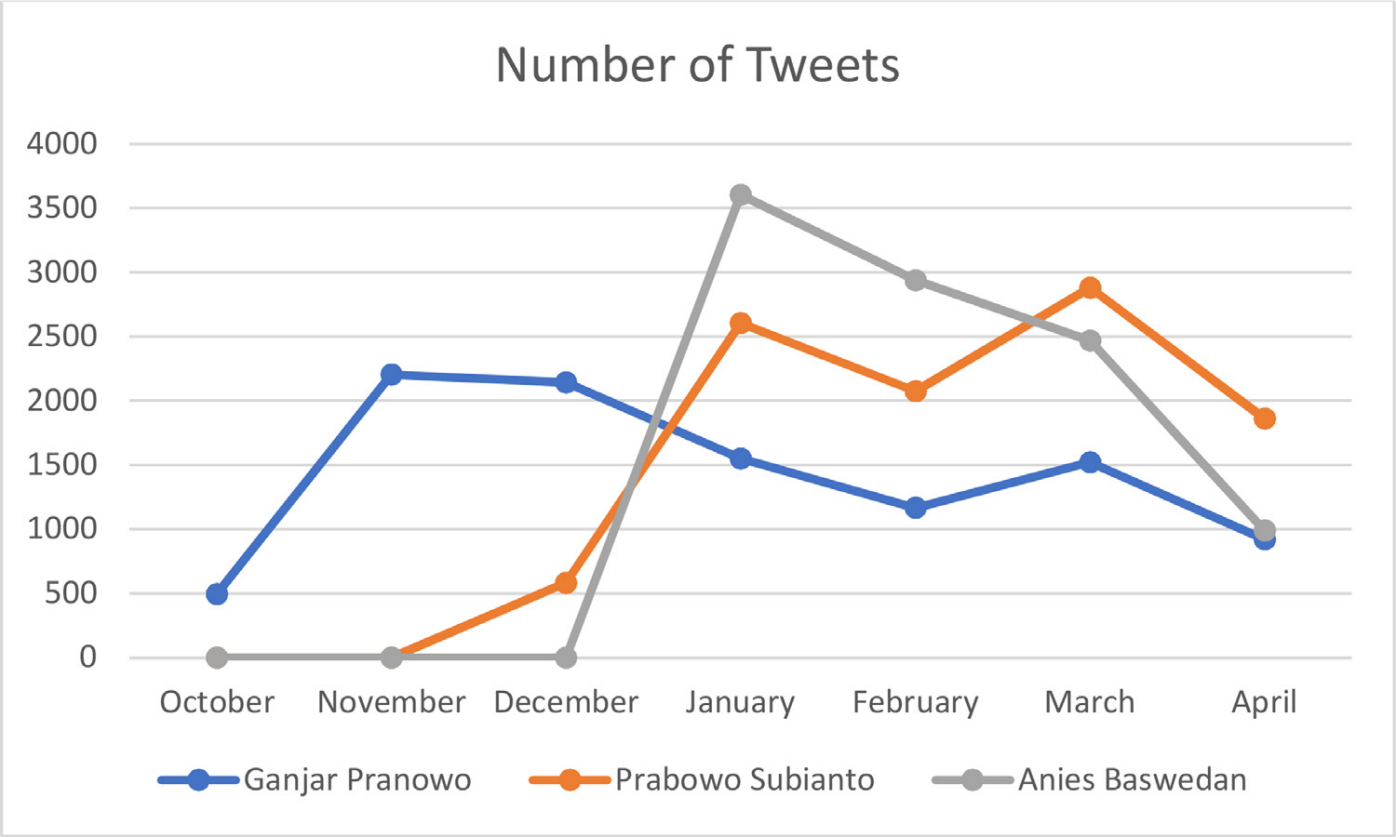
Tweets Discussing Indonesia's 2024 President per month from October 2022 to April 2023.

The data used is in the form of user Tweets based on keywords at the time of data search. The data search used is the name of potential candidates and is followed by “President of Indonesia 2024”, such as “Ganjar Presiden Indonesia 2024”, “Prabowo Presiden Indonesia 2024”, and “Anies Presiden Indonesia 2024” in language. The use of these keywords is intended so that related data can be collected. The time frame for data collection was done in April 2023. Each candidate has a different number of Tweets to reach 10,000 data in each candidate as shown in Fig. 1.

Fig. 1 shows the number of Tweets discussing Indonesian Presidential candidates, Ganjar Pranowo from October 2022 to April 2023 reached 10,000 Tweets, Prabowo Subianto from December 2022 to April 2023 reached 10,000 Tweets, and Anies Baswedan from January to April 2023 reached 10,000 Tweets. Although Tweets consist of several attributes that contain different information, the data retrieval used in this study is only for a few attributes as shown in Table 1.

**Table 1:** Feature item description Twitter.

Item	Description
Tweet Date	Date the tweet was posted on Twitter
Created Account	The dated user joined Twitter
User ID	Number of user ID
Following and Followers	Number of accounts followed and who followed
Tweet Count	Number of posts on Twitter
Tweet Location	Name of the location where the Tweet was posted
Tweet	The Twitter post

These attributes are common, but proving some significant research on this subject requires additional attributes beyond those already acquired. In the utilized data, the obtained information also includes the attribute “username”. However, the attribute is removed before publication to protect the privacy of Twitter users. Social media-based research using a sentiment analysis approach can use the attributes that have been provided as analysis material.

This original data folder contains Tweets as they were originally obtained, using Indonesian. There are no other changes available in this folder from the original data obtained other than the removal of the username attribute.

b. labeled data (Data Folder: labeled data)

Many labeling techniques can be used in Python, but labeling can also be done manually. Manual labeling proves to have better accuracy compared to Python libraries because the annotation technique which was given by humans takes deeper terms [4]. The labeling used in this study is manual with the help of experts so that its accuracy is validated. Indonesian linguists are more trusted to analyze texts for sentiment labeling using Indonesian. Therefore, this study tried to use file labeling in Indonesian when given labeling by linguists. However, in the repository file provided, we have changed it to English to make it easier for readers.

c. cleaned data (Data Folder: cleaned data)

The data goes through preprocessing stages to clean the data, such as case folding, tokenizing, stop word removal, normalization, stemming, and finally, dropping duplicates to eliminate identical Tweets (spam) and empty Tweets. Empty tweets occur after going through preprocessing stages such as URL deletion, tags/symbols, hashtags, and so on. The initial data for each candidate is 10,000, but after these stages, the number of Tweets for each candidate decreases as shown in Table 2.

**Table 2:** Number tweet of candidates.

Candidate	Number of Tweets	NaN	Spam
Anies Baswedan	8,857	66	1,081
Ganjar Pranowo	7,836	115	2,049
Prabowo Subianto	6,758	88	3,155

## 4 Experimental Design, Materials and Methods

### 4.1 Data collection, labeling & preprocessing

**Fig. 2:**
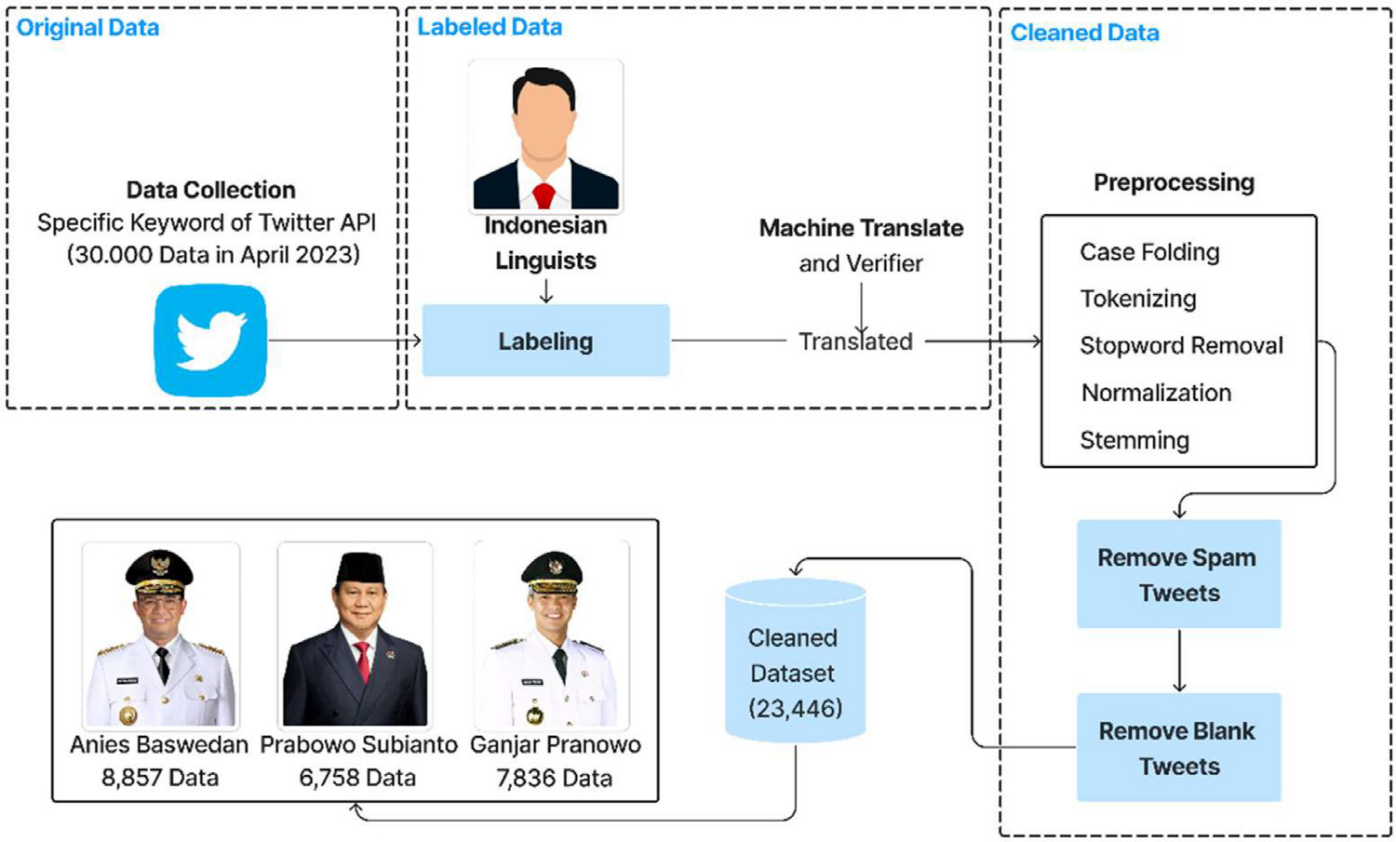
Data collection to label workflows.

Data collection was conducted in April 2023 using the Twitter API. Before collecting data, users must have a developer account. The obtained data is in CSV and Excel formats. The original data is stored in the available repository (Folder: original data). The next step involves the labeling process with the assistance of language experts. This stage is crucial, especially in research on sentiment analysis. Since the tweets collected are in the Indonesian language, the labeling process is also conducted in Indonesian. However, the repository folder is labeled in English (Folder: labeled data). Each tweet is categorized into positive or negative sentiment classes by language experts. Translation into English is performed after the labeling process using machine translation and validation. After translation, the data undergoes preprocessing steps such as case folding, tokenizing, stop word removal, normalization, and stemming to clean the raw data using Jupyter Anaconda tools and the Python programming language (Folder: cleaned data). Data is then filtered to remove repeated (spam) tweets and captured tweets containing empty cells. After completing all the steps, the accumulated data amounts to 23,446, as indicated in Fig. 2.

### 4.2 Experiment

The experiment was conducted as a model for the data that had been processed in the study. This study determined the model using two classification methods, namely Naïve Bayes and Support Vector Machine (SVM) with four kernels. The Naïve Bayes method has been widely used in sentiment analysis [5] because it has the highest accuracy [6] and is applied to many applications [7], [8], [9]. The SVM method is also superior to other classification methods such as research [10,11] in the sentiment analysis approach [12]. The determination of the model was used using three data ratios, namely 70:30, 80:20, and 90:10.

**Fig. 3:**
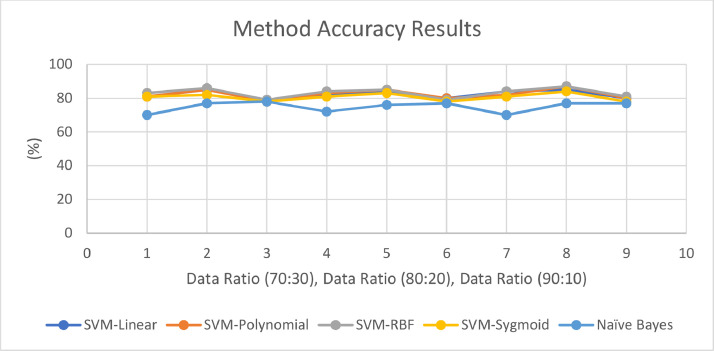
Method accuracy results curve.

Data that has been labeled will go through the extraction stage of the TF-IDF (Term Frequency - Inverse Document Frequency) feature to calculate the amount of weight in a document, the more relevant important words indicate that the greater the weight owned. Furthermore, classification is carried out using two methods as shown in Table 3. It is shown that these two methods used have an average of above 75 %. SVM has a higher accuracy average than Naïve Bayes in the data used as shown in Fig. 3.

**Table 3:** Comparison of model accuracy on classification methods.

SVM Kernels	Candidates	Accuracy (%)		
		Ratio 70:30	Ratio 80:20	Ratio 90:10
SVM - Linear	Ganjar Pranowo	81	82	84
	Prabowo Subianto	85	84	85
	Anies Baswedan	79	80	80
SVM - Polynomial	Ganjar Pranowo	81	83	82
	Prabowo Subianto	85	85	87
	Anies Baswedan	78	80	80
SVM - RBF	Ganjar Pranowo	83	84	84
	Prabowo Subianto	86	85	87
	Anies Baswedan	79	79	81
SVM - Sigmoid	Ganjar Pranowo	81	81	81
	Prabowo Subianto	82	83	84
	Anies Baswedan	78	78	78
Naïve Bayes	Ganjar Pranowo	70	72	70
	Prabowo Subianto	77	76	77
	Anies Baswedan	78	77	77

### 4.3 Discussion

The data available in the repository is dehydrated by removing the attribute column for the username. This is intended to follow the ethics available on the Twitter platform. In addition to the deletion of the username attribute column, the data available in the repository is no longer deleted because it is useful for analysis in other studies that use this data.

Initial testing of the data was obtained using two text classification methods, namely Support Vector Machine with four kernels and Naive Bayes. Both of these methods are used because they have a good degree of accuracy when faced with text classification. Text data obtained on the Twitter platform is then carried out a classification model using both methods. It was found that the SVM method had better performance calculated based on average accuracy than the Naïve Bayes method. However, the SVM method has a longer computational time than Naive Bayes which does it quickly. Naive Bayes have lower accuracy because they require only small amounts of data [13], this is in contrast to SVM. This proves that the SVM method can be more suitable for data models related to the Presidential election and social media-based text classification. Meanwhile, based on the dataset used, the average dataset of each candidate has fairly good accuracy in the data used today. So the data obtained today can be a recommendation for future research on the Indonesian Presidential election based on user Tweets.

## Limitations

Several bullets describe the limitations of the data acquisition in question.

• Data retrieval is limited to a limited number of attribute items on the Twitter entity through the specific keywords used.
• The collected data is then analyzed in the form of labeling positive or negative sentiment classes. This is obtained from linguists/linguists as partners in research.

## Ethics Statement

Data is collected using Twitter's APIs and filtered to conform to policies established by the Twitter platform [14]. In discussions, we process data and do not mention privacy information to protect Twitter users and ensure anonymity.

## CRediT Author Statement

**Asno Azzawagama Firdaus**: Conceptualization, Methodology, Writing – Original draft, Writing - review & editing, Data curation, Formal analysis; **Anton Yudhana**: Funding acquisition, Project administration, Writing – review & editing, Conceptualization; **Imam Riadi**: Writing – review & editing, Visualization, Conceptualization; **Mahsun**: Resource, Validation, Data curation.

## Data Availability

Indonesia Presidential Candidate's Dataset, 2024 (Original data) (Mendeley Data)
